# Supplementary material for: treekoR: identifying cellular-to-phenotype associations by elucidating hierarchical relationships in high-dimensional cytometry data
Source: Genome Biol. 2021 Nov 29;22:324. doi: 10.1186/s13059-021-02526-5 (PMC8628061; doi:10.1186/s13059-021-02526-5)
Supplement: Supplementary file 1 — Additional file 1. Supplementary materials [42, 43]. [file 13059_2021_2526_MOESM1_ESM.docx]

**Supplementary**

### **Comparison of use of t-test in treekoR to count models edgeR and GLMM**

## In this section, p-values obtained from using t-tests on proportions are compared to p-values obtained from using edgeR and GLMM on counts. P-values were computed from testing for association with COVID-19 response using a flow cytometry sample T Cells[^4^](https://paperpile.com/c/dKyHE7/CQJUW). When the p-values from treekoR were compared to the p-values from edgeR and GLMM, Spearman correlations of 0.895 and 0.838 (respectively) were obtained using %total, and 0.862 and 0.895 (respectively) were obtained using %parent (**Figure S1**). Although there are differences between how the methods handle the variability in the data, leading to different p-values in some cell types, the p-values obtained from modelling cell types as counts and proportions broadly followed the same trend.

### **Different hierarchical clustering methods uncover varying %parent relationships in cytometry data**

In this section several tree structures derived from different hierarchical aggregation techniques are compared, in addition to a manual gating tree structure. The manner in which these ultimately result in capturing different signals from the data are demonstrated. Although no single representation may be the absolute correct one, exploring these different representations can begin to help analysts to uncover a broader scope of complex relationships that exist within cytometry data to discover the cellular heterogeneity between patient samples.

In our framework, the HOPACH algorithm was used to hierarchically aggregate the clusters into a tree. HOPACH is a clustering algorithm that was originally developed for gene expression data analysis, but has useful properties for the analysis of high-dimensional cytometry data. One advantage is the lack of restriction for splits to be binary, allowing up to 15 child nodes per node (**Figure S2a**). In this example it can be observed that Pre-B cells, Mature B Cells and Immature B Cells fall under the same parent node, which, for example, would allow analysts to determine whether the compositional makeup of B cells plays any role in patient disease. Another advantage of HOPACH is the cluster collapsing step, which helps to alleviate any incorrect splitting of clusters. This helps prevent the tree from containing too many branches, which can reduce some correlations between proportions as our framework explores each of the parent-child relationships in the generated trees.

The high-dimensional nature of single cell cytometry data gives rise to numerous biologically relevant cell type hierarchies. treekoR acknowledges this by providing a framework which is not restricted to one specific cell type hierarchy constructed by a specific algorithm. We compared trees constructed using Hierarchical Ordered Partitioning And Collapsing Hybrid (HOPACH)[^17^](https://paperpile.com/c/dKyHE7/c6gx6) clustering, average-linkage hierarchical clustering, and single-linkage hierarchical clustering via ‘tanglegrams’ (a pair of trees drawn with edges connecting matching leaves between the pair) on a PBMC sample from a healthy bone marrow donor[^42^](https://paperpile.com/c/dKyHE7/FWiNz) (**Figure S2b-S2d**). The comparison highlights distinct cell type trees, which would consequently result in distinct quantifications of %parent proportions. Average-linkage hierarchical clustering - used in algorithms such as treeclimbr and citrus - and single-linkage clustering - closely resembling minimum spanning trees[^43^](https://paperpile.com/c/dKyHE7/p9iOu) used in SPADE and visualising FlowSOM - generate distinct hierarchical representations of the data. In this dataset, HOPACH provided a representation more closely resembling the manual gating tree constructed by Bendall et al. Of importance is that one representation of cell type hierarchy may not necessarily be the most informative, but each of these representations can lead to diverse yet relevant %parent relationships.

When comparing the HOPACH constructed tree to the manual gating hierarchy, a clear difference is how NK cells and GMP cells are grouped - NK and GMP fall under the same immediate parent node in the HOPACH tree whilst they are not in the gating tree (**Figure S2b**). An analyst may gate the cell types differently using two markers such as CD34 & CD45RA (**Figure S2f**). Although this provides a more interpretable representation to cell groupings, it is clear in the t-SNE plot (**Figure S2g**) how they would group together via clustering which considers all the available markers in unison. This indicates the effect of the experimental panel design, where some biologically distinct cell subpopulations can group together in automated clustering methods when there are more markers to distinguish them. Although there are notable differences between the automatically generated tree and the manually gated hierarchy, the HOPACH clustering is able to regenerate some of the cell type groupings. Despite some scenarios where the parent proportions may make less sense, this provides our framework with a big advantage over manual gating through being more efficient in handling larger datasets as well requiring a lower extent of prior knowledge to subset cells.


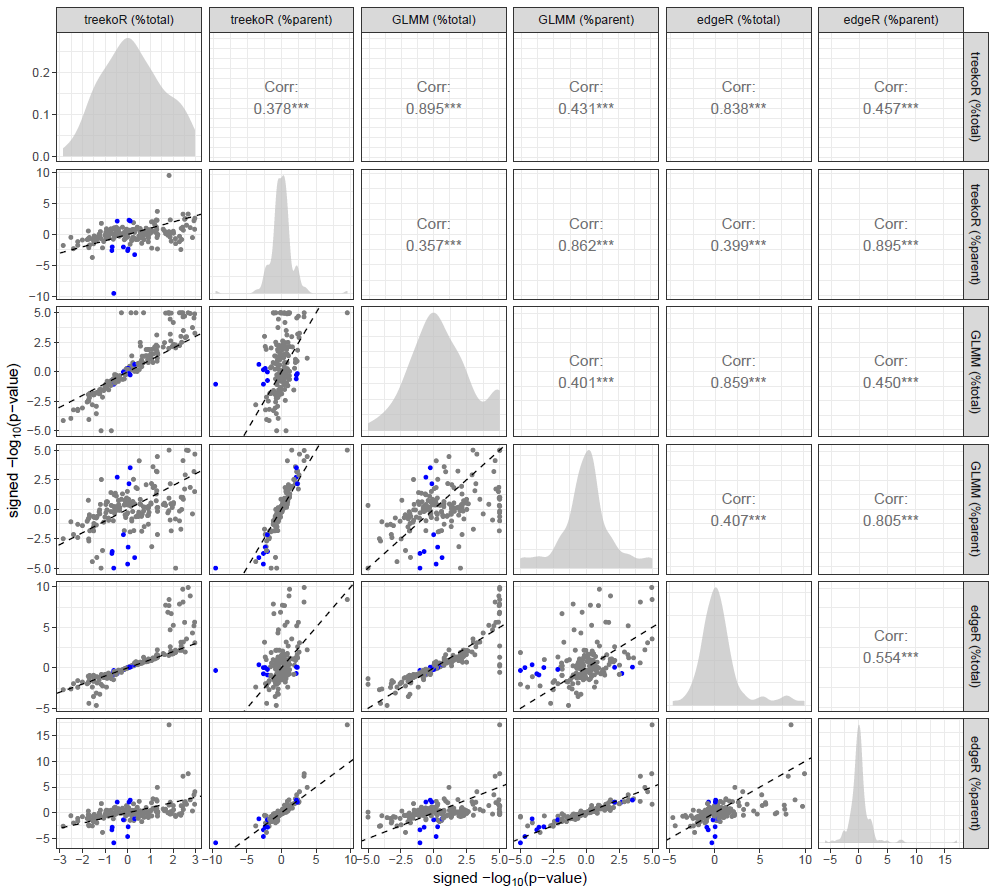


**Fig S1: A pairs plot of p-values generated from testing using counts in edgeR, counts in GLMM and proportions in t-tests.**  Each point in the scatterplots represents a cell type obtained from running treekoR on the dataset COVID-19 T Cells (CD8+ T cell compartment), and their respective p-values from testing between healthy vs COVID-19. The negative log10 of the p-values multiplied by the sign of the log fold change were plotted on the x and y axes in the scatterplots. Points highlighted in blue represent cell types which had a p-value < 0.01 from a t-test using %parent and p-value > 0.1 from a t-test using %total. Spearman correlations are shown in the upper triangle of the plot grid.


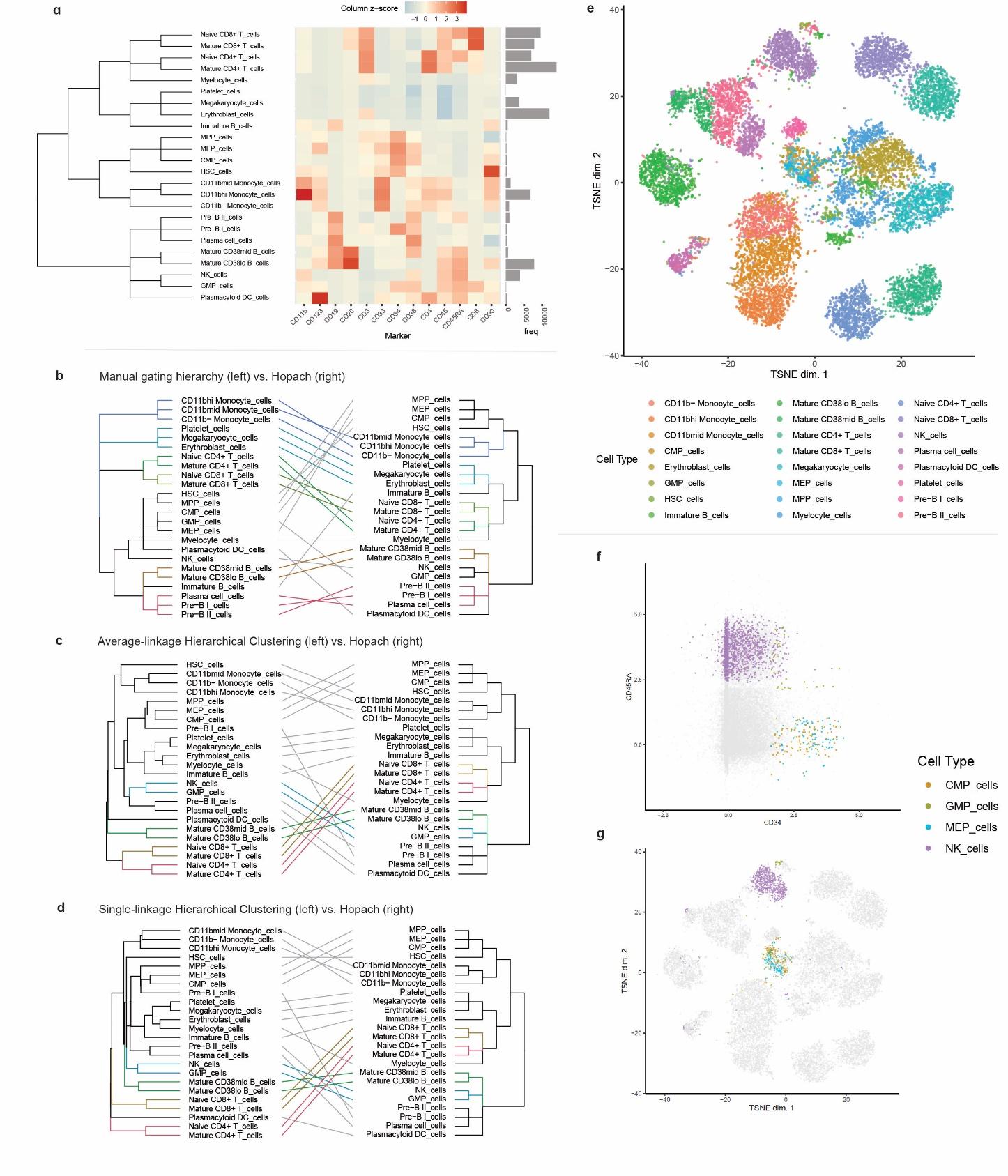


**Fig S2: treekoR facilities varying cell type hierarchies to enable the measurement of cell types %parent** **a.** A heatmap of the median marker frequencies, clustered using HOPACH, for each cell type as subsetted with manual gating from a healthy human bone marrow sample[^42^](https://paperpile.com/c/dKyHE7/FWiNz) (top left). **b.**Tanglegrams comparing two trees to highlight differences in cell type hierarchies: manual gating hierarchy vs. HOPACH clustering; **c.** average-linkage hierarchical clustering vs. HOPACH clustering; and **d.** single-linkage hierarchical clustering vs. HOPACH clustering (bottom right). **e.** A t-SNE plot of the sample, highlighted by the manually gated cell types **f.** Scatterplot of CD34 vs. CD45RA with only CMP, GMP, MEP & NK cells highlighted **g.** A t-SNE plot of the sample with only CMP, GMP, MEP & NK cells highlighted
