# Supplementary material for: treekoR: identifying cellular-to-phenotype associations by elucidating hierarchical relationships in high-dimensional cytometry data
Source: Genome Biol. 2021 Nov 29;22:324. doi: 10.1186/s13059-021-02526-5 (PMC8628061; doi:10.1186/s13059-021-02526-5)
Supplement: Supplementary file 2 — Additional file 2. Review history. [file 13059_2021_2526_MOESM2_ESM.docx]

Review History

**First round of review**

**Reviewer 1**

**Were you able to assess all statistics in the manuscript, including the appropriateness of statistical tests used?**

Yes: The statistics here are simply 2-sample t-tests. I've asked a question about this in my review.

**Were you able to directly test the methods?**

No

**Comments to author:**

Chan et al. present treekoR, a method and Bioconductor package to conduct differential abundance tests (comparing experimental conditions) for single cell datasets, typically those from CyTOF or even single cell RNA-seq. The importance aspect that they argue for is to make differential abundance comparisons not necessarily relative to the full set of cells (% total), but relative to a smaller subpopulation (% parent); in this case, the proposed subpopulation to compare against are the "parents", given a hierarchical clustering of cells. HOPACH clustering gives some extra nuance, such that a parent node can include multiple offspring nodes. They use several datasets to make the case for this and I find it quite convincing. Overall, the paper is well organized, a pleasure to read and the authors already have a Bioconductor package for the approach. I only have a small number of queries or suggestions:

1. What tests are used? I didn't find too much discussion about the motivation for it, but I presume the authors are simply using a two sample t-test in all cases on the proportions (% total OR % parent). But these are count data and while in large samples, this probably makes no difference, I would've expected that count models were used to account for the uncertainty in the proportions. At a minimum, I would expect some discussion about why they are using simple t-tests and if interesting, it would be also good to see how much difference it makes.

2. Discreteness of parent populations. In reading the first half of the paper, I kept wondering what is a good reference population to compare against. The authors argue quite convincingly that '% total' is not always most appropriate and I fully agree. Now, the question is: is '% parent' really the best reference? Here, it depends on the clustering. I think HOPACH is a good first approximation, but there could also be a bit more flexibility here .. maybe the parent of the parent is the better reference population to compare against (e.g., all T cell subsets). Then, I can't help but think of approaches like treeclimbR that search the space comprehensively (here, I'm thinking about the parent space to be more comprehensively searched, instead of what treeclimbR does). The main question is: is it worth looking beyond the immediate parent?

3. I can't help but think of *simulating* certain cases to show that available approaches miss important biological changes. Have the authors thought about this or is it too complex to construct?

4. I suggest to clean up the analysis repository (https://github.com/adam2o1o/treekoR_analysis) in the following ways: 1. build all RMDs to HTML (include both!) and also include a sessionInfo() in the interest of reproducibility (at least gives indication of what package stack is used); 2. check that all datasets are on github (in the script, there are datasets referred to that are not in the github repo, e.g., "data/sce_tomic_cmv_gated.RData"); 3. since github repos may change and/or not last forever, I suggest to take a snapshot and put zenodo DOI in the manuscript (https://guides.github.com/activities/citable-code/).

**Reviewer 2**

**Were you able to assess all statistics in the manuscript, including the appropriateness of statistical tests used?**

Yes: Yes! Appropriate statistical methods were applied.

**Were you able to directly test the methods?**

No

**Comments to author:**

Chan et al address the problem routinely faced when analysing single cell data with respect to end points: to represent cell subset quantities either as a percentage of their parent population (akin to manual gating), or instead as a percentage of the total cell count (as obtained by default from clustering).

Contributions of Chan et al are twofold:

[1] METHODS: Chan and colleagues provide a flexible and intuitive pipeline in R, -released as a fully documented package on bioconductor & github-, to create both these representations of cell subsets. Importantly, data used for the paper is traceable and hosted on their github page along with the code to recreate figures from the paper.

[2] BIOLOGY: Chan and colleagues thoroughly benchmark these two alternative ways of representing cell subset quantities using 11 single cell datasets of various types (flow, Cytof, single cell RNAseq), and outcomes (COVID, cancer, B-cell activation) etc), in various analytic settings (association & prediction) to show that both representations seem to yield complementary results. Importantly, they show that it is hard to predict beforehand which representation seems most suitable, AND that some cell subsets can only be identified using either one or the other representation.

VERDICT: Overall I find this manuscript well-written, to-the-point, and appealing to a broad readership dealing with the analysis of single cell data. I would strongly advice for publication of the manuscript as is.

Kind regards,

Erik van den Akker

**Authors Response**

**Point-by-point responses to the reviewers’ comments:**

We appreciate the reviewers’ thoughtful comments on our initial submission. We have responded to each of these comments in detail below.

Reviewer #1: Chan et al. present treekoR, a method and Bioconductor package to conduct differential abundance tests (comparing experimental conditions) for single cell datasets, typically those from CyTOF or even single cell RNA-seq. The importance aspect that they argue for is to make differential abundance comparisons not necessarily relative to the full set of cells (% total), but relative to a smaller subpopulation (% parent); in this case, the proposed subpopulation to compare against are the "parents", given a hierarchical clustering of cells. HOPACH clustering gives some extra nuance, such that a parent node can include multiple offspring nodes. They use several datasets to make the case for this and I find it quite convincing. Overall, the paper is well organized, a pleasure to read and the authors already have a Bioconductor package for the approach. I only have a small number of queries or suggestions:

1. What tests are used? I didn't find too much discussion about the motivation for it, but I presume the authors are simply using a two sample t-test in all cases on the proportions (% total OR % parent). But these are count data and while in large samples, this probably makes no difference, I would've expected that count models were used to account for the uncertainty in the proportions. At a minimum, I would expect some discussion about why they are using simple t-tests and if interesting, it would be also good to see how much difference it makes.

*Response: The reviewer is correct, we used a two sample t-test in all cases. As suggested, due to 1) the large cell counts seen in many cytometry datasets and 2) the large number of samples used, t-tests or wilcoxon rank sum tests are often used due to their simplicity.. We have updated the manuscript to now acknowledge that count models could be used too (Page 6 Line 18). We adapted the count models that were used in the diffcyt (Weber et al.) workflow, including generalized linear mixed models (GLMM) and edgeR. We have included a comparison of the p-values generated using these count models and the ones generated via a t-test in Additional File 1: Figure S1. Whilst there are minor differences between the p-values generated from the different approaches, the key findings, particularly when comparing %parent to %total do not change.*

2. Discreteness of parent populations. In reading the first half of the paper, I kept wondering what is a good reference population to compare against. The authors argue quite convincingly that '% total' is not always most appropriate and I fully agree. Now, the question is: is '% parent' really the best reference? Here, it depends on the clustering. I think HOPACH is a good first approximation, but there could also be a bit more flexibility here .. maybe the parent of the parent is the better reference population to compare against (e.g., all T cell subsets). Then, I can't help but think of approaches like treeclimbR that search the space comprehensively (here, I'm thinking about the parent space to be more comprehensively searched, instead of what treeclimbR does). The main question is: is it worth looking beyond the immediate parent?

*Response: This comment resonates with us and is something we had put thought into as we were developing treekoR. We believe it has largely been addressed in the discussion paragraph on Page 11 Line 6. In short we believe it is worth looking beyond the immediate parent, which is one of the reasons we were attracted to HOPACH clustering. If the number of cell-types remains “small” (<100) we can imagine an exhaustive search being possible but do not feel equipped to offer a sensible solution for accounting for multiple highly correlated hypothesis tests. We see this as an exciting avenue for future research and collaboration.*

3. I can't help but think of *simulating* certain cases to show that available approaches miss important biological changes. Have the authors thought about this or is it too complex to construct?

*Response: It is difficult to generate an adequate simulation that is not overly complex and depicts the instances in an easy to understand/digest manner. Complex simulations did not give more clarification or justification beyond our evaluation using twelve real data sets.*

4. I suggest to clean up the analysis repository (https://github.com/adam2o1o/treekoR_analysis) in the following ways: 1. build all RMDs to HTML (include both!) and also include a sessionInfo() in the interest of reproducibility (at least gives indication of what package stack is used); 2. check that all datasets are on github (in the script, there are datasets referred to that are not in the github repo, e.g., "data/sce_tomic_cmv_gated.RData"); 3. since github repos may change and/or not last forever, I suggest to take a snapshot and put zenodo DOI in the manuscript (https://guides.github.com/activities/citable-code/).

*Response: The github has been updated with built RMDs including session information in each. The datasets are available in github except for the “data/sce_tomic_cmv_gated.RData” as the file is too big. As such, we have provided a link to the original CyTOF .fcs files and all preprocessing steps to reproduce the analysis from the file “treekoRAnalysis_tomic_cmv.rmd”. A zenodo was also created for the git repository and referenced in the manuscript*
